# Validity and measurement invariance of the Unified Multidimensional Calling Scale (UMCS): A three-wave survey study

**DOI:** 10.1371/journal.pone.0209348

**Published:** 2018-12-14

**Authors:** Michelangelo Vianello, Anna Dalla Rosa, Pasquale Anselmi, Elisa Maria Galliani

**Affiliations:** 1 Department of Philosophy, Sociology, Education and Applied Psychology, University of Padova, Padova, Italy; 2 Department of Political and Juridical Sciences and International Studies, University of Padova, Padova, Italy; University of the West Indies at Saint Augustine, TRINIDAD AND TOBAGO

## Abstract

The accumulation of scientific knowledge on calling is limited by the absence of a common theoretical and measurement framework. Many different models of calling have been proposed, and we do not know how much research results that refer to a specific model are generalizable to different theoretical accounts of calling. In this article, we investigate whether two leading models of calling tackle the same construct. The two models were merged into a comprehensive framework that measures calling across seven facets: Passion, Purposefulness, Sacrifice, Pervasiveness, Prosocial Orientation, Transcendent Summons, and Identity. We then developed the Unified Multidimensional Calling Scale (UMCS) drawing from previous published items. Across two surveys involving college students (N = 5886) and adult employees (N = 205) the UMCS was proved to be valid and reliable. We also observed that the UMCS is invariant across time and calling domains. Finally, we found that facets of calling have very different relationships with outcomes and concurrent measures, suggesting that results obtained with a smaller set of facets are not generalizable to the higher-order construct of calling or to a different model that does not share the same facets.

## Introduction

Research on career calling has grown exponentially, and a number of theoretical and measurement models have been proposed. Yet, differences across models of calling limit the generalizability of results across studies [1, 2, 3]. Currently, we do not know how much research results observed with a specific model of calling are generalizable to other theoretical accounts. What may be true for one conceptualization of calling may not be true for the others. For instance, predictors of a meaningful passion for a domain may not be predictors of a transcendent summons for that domain, although they are both key dimensions of different models of calling [4, 5]. Heterogeneity in measures and conceptualizations of the same construct may lead to contradictory results. In this article, we propose a multidimensional model that combines the two most common theoretical accounts of calling, and present an extensive construct validation of the resulting scale (Unified Multidimensional Calling Scale, UMCS). We investigate its first and second order structure, and test whether the second-order model is invariant across measurement occasions and calling domains. Finally, we investigate whether facets of calling are differently related to three outcomes and two concurrent measures.

### Theoretical background

In general, there seems to be agreement among researchers that calling is multidimensional, but there is no consensus on its components. Dik and Duffy [6] defined calling as a transcendent call to pursue a career, whereas others see it as a passion for a domain [3, 5, 7, 8, 9] that motivates people to engage in related activity [8, 9], and to sacrifice other areas of life to live out their calling [5, 7]. According to some authors, having a calling pervades all the dimensions of life because it is part of one’s identity, defines who a person wants to be [3, 5, 9], and gives meaning and purpose to life [5, 6, 8].

In the last ten years, theories on calling developed around two main approaches [2]: The neoclassical and modern approaches. The former emphasizes the importance of a transcendent call and prosocial duty [6, 7, 10, 11]. In line with this approach, Dik and Duffy (2009) defined calling as the experience of a summons from an external source (for instance God, community, family) to do a work that provide meaning and contributes to the common good. The modern approach does not consider an external or transcendent source of calling neither a prosocial orientation; on the contrary, it emphasizes the subjective nature of calling and focuses on an inner drive toward self-fulfillment [5; 8]. Conforming to this conceptualization, Dobrow and Tosti-Kharas [5] and Dobrow [12] define calling as a consuming passion towards a specific meaningful work that pervades all dimensions of individuals’ life and contributes to individuals’ personal and professional identity. Both the neoclassical and modern approaches associate calling with individuals’ willingness to make sacrifices [5, 7]. With the aim of building a comprehensive model of calling, we identified seven facets that are recurrent across theories and that represent both neoclassical and modern approaches: identification with the calling domain, pervasiveness of thoughts regarding the calling domain, purposefulness, transcendent summons, prosocial orientation, sacrifice, and passion. Table 1 summarizes the main components of calling across six different theoretical accounts.

**Table 1:** Dimensions of calling across six different theoretical accounts.

	Dobrow & Tosti Kharas (2011)	Dik, Duffy et al. (2009, 2012)	Wrzesniewski et al. (1997)	Bunderson & Thompson (2009)	Praskova et al. (2015)	Hagmeier & Abele (2012)
Identification	√		√			√
Pervasiveness	√		√			
Purposefulness	√	√			√	
Transcendent summons		√		√	√	√
Prosocial orientation		√	√		√	√
Sacrifice	√					
Passion	√		√		√	

The *identity* dimension [13] is present in Dobrow and Tosti-Kharas [5] and in Wrzesniewski and colleagues’ work ([9] p24): “What [one] does for living is a vital part of who [one] is”. Individuals experiencing a strong calling “feel their involvement in the calling domain is central to their identity” ([5] p1005) and part of their destiny [12]. These models of calling also include a *pervasiveness* dimension: “For people who have a calling, one’s work domain is continuously present in one’s consciousness” ([12] p4). The *purposefulness* dimension is present in Dik and Duffy [6], Dobrow and Tosti-Kharas [5], and Praskova and colleagues [8]: Calling gives meaning to one’s life, and helps to derive a precise sense of purpose or meaningfulness. According to Dik and Duffy ([6] p427), calling is “*a transcendent summons* that is experienced as originating beyond the self” and that has a *prosocial* purpose: It is an approach to a life role that “holds other-oriented values and goals as primary sources of motivation”. The Transcendence and Prosocial Orientation dimensions are both present in Bunderson and Thompson [7], and Hagmeier and Abele [3]. The Prosocial Orientation dimension is also present in Wrzesniewski and colleagues [11]. Many authors agree that people who have a calling are pushed to answer it [7, 8, 11]. Hence, they are likely to *sacrifice* time, energy, and money to pursue their calling. Lastly, the most common dimension across conceptualizations is that of *passion*: People with a calling feel a deep enjoyment and satisfaction when they are involved in activities related to their own calling [3, 5, 7, 8, 11].

## Materials and methods

### Participants

The first sample was composed of Italian college students. The second sample of Italian working adults. The first sample was longitudinal: data were collected in 3 waves using a non-experimental online survey. The second and third wave respectively occurred 12 and 24 months after the first data collection. 5,886 Italian college students were involved in the first data collection, 1,700 students were involved in the second data collection, and 881 took part to the third data collection. A sample of 434 students participated at all the three waves (7.37% of the initial respondents). Results of sample attrition analysis across the three waves of data collection, which are provided in a supplement (https://osf.io/bw234/download), suggest that the only non-trivial difference between stayers and leavers concerns participants’ age at Time 1. Students who provided data at all three observations are younger (21.97) than students who drop out the data collection (23.49) before Time 3. This has occurred because older students were more likely to graduate. After graduation, their institutional e-mail is automatically de-activated.

The sample was mainly composed of women (63.8%, 65.8%, and 68% females at Times 1, 2, and 3, respectively). Participants’ age ranged between 18 and 69 (*M*_T1_ = 23.37; *SD*_T1_ = 5.39; *M*_T2_ = 23.47; *SD*_T2_ = 4.82; *M*_T3_ = 24.02; *SD*_T3_ = 4.50). At the time of the three data collections, participants were active bachelor or master students enrolled in 24 different study domains. The working adults sample (*N* = 205) was composed of 106 public high school teachers and 99 public and private employees who completed a self-administered paper-and-pencil questionnaire. This sample was mainly composed of women (67.3%) and participants’ age ranged between 20 and 78 (*M* = 46.81; *SD* = 11.18). Participants’ seniority in their organizations ranged from 0 to 48 years (*M* = 19.18; *SD* = 11.69).

College students were invited to take part to the survey via e-mail. Upon receiving the invitation message, participants were informed that participation in the survey presented no risk, that the data produced would have been anonymized and used exclusively in aggregated form for scientific purposes, and that they could have withdrawn at any time without giving a reason. Participants were then informed that going further in the survey would have been interpreted as their signature on the consent form. Their consent was electronically recorded. The research has been approved by the Italian Ministry of Education, University and Research as part of a larger project. Data protection followed regulation of the Italian country (Legislative Decree n. 196/2003) and of the EU regulation n. 2016/679. Participants voluntarily participated in the research. Participants in the first sample were offered a €25 and €15 lottery incentive, respectively at T2 and T3, awarded to 50 and 70 randomly chosen students. Participants in the second sample did not receive any rewards.

### Measures

#### Calling

Items were taken from the two most popular scales of calling: 22 items were extracted from the calling scale developed by Dobrow and Tosti-Kharas [5], and from the Calling and Vocation Questionnaire (CVQ) by Dik and colleagues [4]. Dobrow and Tosti-Kharas [5], provided a multidimensional definition of calling consisting of four facets (passion, sacrifice, pervasiveness and identity), but developed a unidimensional scale of 12 items. We added a thirteenth item regarding sacrifice to balance the number of items across facets. The CVQ [4] is composed by 24 items and measures the presence of and search for a calling with three subscales: Transcendent summons, purposeful work and prosocial orientation. We selected nine items from the presence of a calling scale with the highest loading in their corresponding factors. When necessary, items’ wording was adapted to the specific samples involved in this research. For example, the item “I am passionate about playing my instrument/singing/engaging in my artistic specialty/business/being a manager” [5] was modified in: “I am passionate about what I am studying” for the sample of students, and in “I am passionate about teaching/my work” for the sample of teachers/workers. The 22 items were translated and back-translated by three independent experts. As regards the students sample, all analyses reported in this article are based on the responses collected at Time 2 (*N* = 1,700), except for the longitudinal invariance test, for which data from all the three waves were used. The wording of seven items was modified after the first wave. For the longitudinal invariance test, responses to these items have been recoded as missing in the first wave. Details on item changes across waves and the final version of the scale are available at https://osf.io/csn7f/download.

#### Concurrent measures

Having a vocation was assessed with a single item measure asking students to answer the following question: “How much do you have a vocation for a specific study/work domain?” on a 4-point scale from “not at all” to “very much”. In addition, we assessed calling orientation toward work with a single item developed by Wrzesniewski and colleagues [9]. Students were asked to read the paragraph describing a worker with a calling orientation and to rate how much they identified with the worker’s profile on a 4-point scale from ‘not at all similar’ to ‘totally similar’.

#### Outcomes

Participants’ intention to continue studying was evaluated with one item on a scale from 1 (“I am going to leave my study program”) to 4 (“I am going to finish my study program”). The degree to which students felt they were currently living their calling was assessed with the item: “Are you living out your calling in the program you are enrolled?”. The degree to which participants were satisfied with their study program was measured with the question: “How much are you satisfied with your study program?” Both the items were evaluated on a 4-point scale from ‘not at all’ to ‘very much’.

### Statistical approach

The initial sample of college students was randomly split into two data sets. The first data set (*N* = 881) was used for the exploratory factor analysis (EFA), whereas the second (*N* = 819) was used for the confirmatory factor analyses (CFA). All the analyses were performed using either IBM SPSS 24 or MPlus 6.0 [14, 15].

*Comparison criteria between nested models*. To assess the fit of CFA models to the data and to compare competing models, the chi-square test of close fit, the comparative fit index (CFI), the standardized root mean squared of residuals (SRMR), and the root mean square error of approximation (RMSEA) were adopted. Compared to the null model, acceptable fit was defined by the following criteria: RMSEA ≤ .06, SRMR ≤ .08, CFI ≥ .95 [16]. Competing nested models were tested using the chi-square test of close fit, and differences in CFI, RMSEA, and SRMR. The alpha level of the test of close fit was set to .005 [17]. When the test of close fit was significant, we looked at differences in fit indexes to understand whether changes across groups or measurement occasions were non-trivial. Thresholds for accepting non-invariance were set according to Chen [18]: a change ≥ -.010 in CFI, supplemented by a change ≥ .015 in RMSEA or a change ≥ .010 in SRMR would denote noninvariance. For testing metric invariance, following suggestions by Chen [18], the threshold for SRMR has been set to ≥ .030 since this index is particularly sensitive to changes in factor loadings.

*Testing measurement invariance*. Measurement invariance across time and study domains was tested at four levels [19, 20, 21] by comparing a series of nested models. The four levels of invariance were:

1. *configural* (the same item must load onto the same latent factor),
2. *metric* (equal factor loadings),
3. *scalar* (equal item intercepts and factor means), and
4. *strict* (equal error variances).

To test longitudinal measurement invariance, the autocovariances between the errors of the same indicators were estimated across the three measurement occasions. In the configural and metric model, the intercepts of the first-order factors and the mean of the second-order factor were constrained to zero to achieve identification [22, 23, 24]. To test metric invariance, the first- and second- order factor loadings of the same variables were constrained to be equal across the three measurement occasions or the different study domains. To test scalar invariance, the intercepts of the indicators were constrained to be equal. The identification of the mean structure of the model [22, 24] was accomplished by constraining to zero the second-order factor mean and the first-order factor intercepts in a reference group. Therefore, the latent means in the other groups represent deviations from the reference groups. When both factor loadings and item intercepts are invariant, scores of different groups have the same measurement metric and the same scalar (i.e., the same origin), hence the latent factors can be compared across groups and time points [21, 24]. The strict invariance of the observed variables and first-order factors across time points and groups was then tested.

## Results

### First order structure

We expect the UMCS to be multidimensional, but we do not have enough information to hypothesize a specific factor structure. Hence, we firstly estimated an exploratory principal axis factoring model with oblique Promax rotation on all the items. Kaiser’s [25] eigenvalue rule suggested a 5-factor solution, which accounted for 62.93% of variance. Parallel analysis [26] suggested the presence of 6 factors, which accounted for 66.56% of variance. Finally, the scree test [27] suggested 7 factors accounting for 69.15% of variance. Since these methods did not converge to one solution, we decided to test and compare these models with CFAs. Results, which are reported in Table 2, suggest that the 7-factor solution fits the data better than the 5-factor solution (Δχ^2^(11) = 1195.10, *p* < .001; ΔCFI = -.106; ΔRMSEA = .046; ΔSRMR = .023), and the 6-factor solution (Δχ^2^(6) = 283.96, *p* < .001; ΔCFI = -.025; ΔRMSEA = .013; ΔSRMR = .001). Therefore, the 7-factor solution was selected as the best fitting model and interpreted. Content analysis of items in each factor suggested that the seven facets of calling measured by these items are: Passion, Sacrifice, Transcendent Summons, Prosocial Orientation, Pervasiveness, Purposefulness, and Identity. All item loadings were above .65. We then tested this model (Model 1) on the total sample of students at Time 2 (*N* = 1,700), and found a good fit to the data (χ^2^(188) = 1259.79, *p <* .001, CFI = .96, RMSEA = .06, SRMR = .05). Two cross-loadings from one item (Per_3: “My days would be less meaningful if I was not involved in these studies”) to both the Pervasiveness and Identity factors improved the data-model fit (χ^2^(187) = 1095.937, *p <* .001, CFI = .962, RMSEA = .053, SRMR = .039). Parameter estimates of Model 1 are reported at the following link: https://osf.io/b6mwh/download. Correlations among the seven factors were moderate (*r* = .23) to large (*r* = .73), suggesting that they are correlated but independent [28; 29].

**Table 2:** Fit indexes of first-order factor models.

Number of factors	χ^2^	*df*	CFI	RMSEA	95% CI	SRMR
**5**	1846.99	199	.85	.101	.096 - .11	.069
**6**	935.86	194	.93	.073	.064 - .073	.047
**7**	651.90	188	.96	.055	.05 - .06	.046

### Second order structure

**Fig 1:**
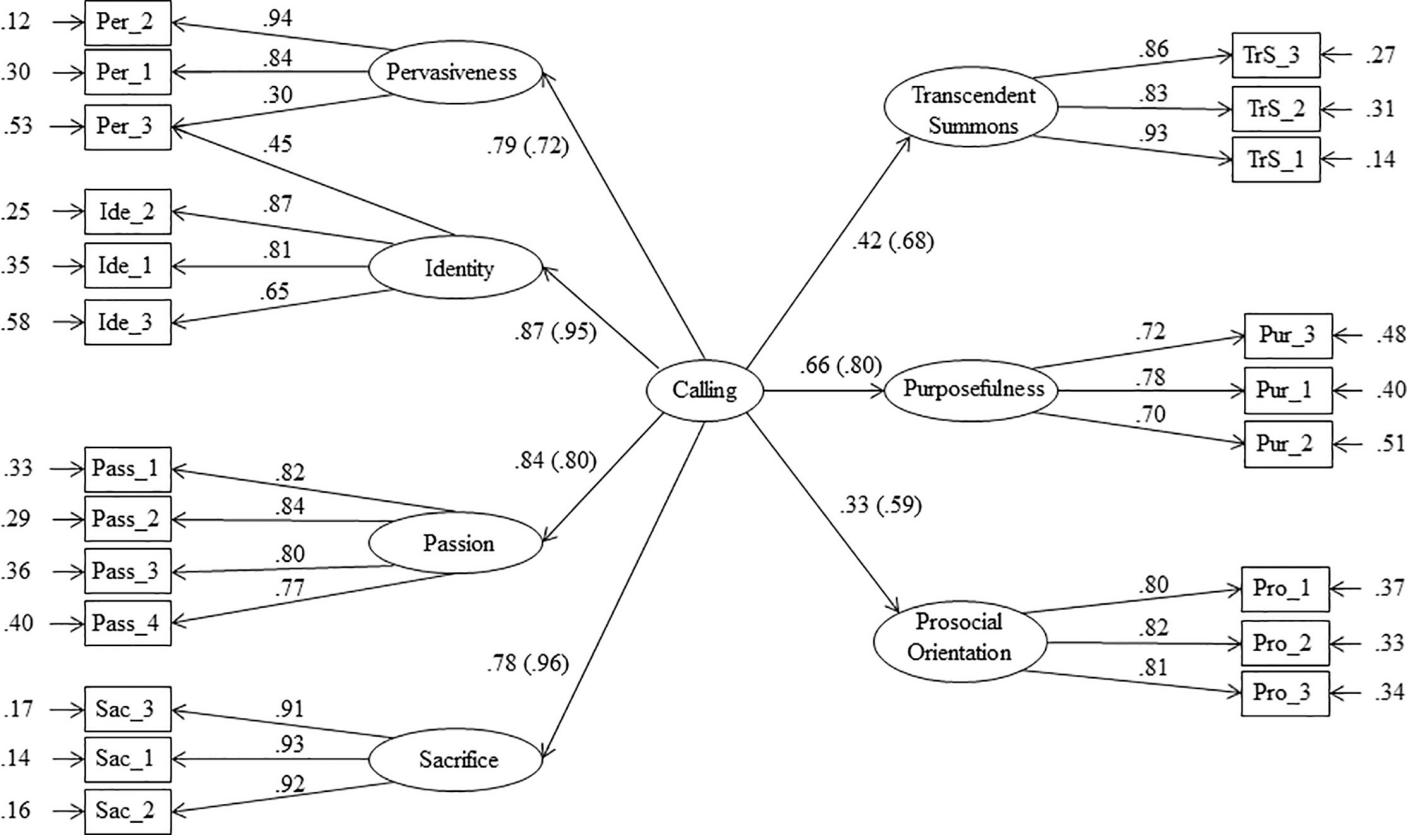
Model 2: The best fitting second order factor model for students. (χ^2^(201) = 1274.24, CFI = .955, RMSEA = .056, 95% CI [.053 - .059], SRMR = .039). Loadings in parentheses are estimated on a sample of working adults.

To investigate whether the seven factors are facets of the same construct, we estimated a second order CFA. The seven-factor model (Model 1) was adopted as a reference. The goal of a second-order CFA was to identify a more parsimonious model applying a covariance structure to the first-order factors. First, we compared Model 1 to a more parsimonious model in which one second order factor (i.e., Calling) was estimated (Model 2: χ^2^(201) = 1274.24, CFI = .955, RMSEA = .056, 95% CI [.053 - .059], SRMR = .039). The chi-square difference test was significant (Δχ^2^(14) = 178.30, *p* < .001), but differences in CFI and RMSEA were trivial (ΔCFI = -.007; ΔRMSEA = .003; ΔSRMR = .011). Hence, Model 2 was chosen for parsimony. In this model, all the second-order factor loadings were statistically significant (*p* < .001), and varied in magnitude from .33 to .87. Identity and Passion had the highest loadings on the second-order factor (respectively, β = .87, β = .84). Prosocial Orientation and Transcendent Summons presented the smallest loadings (respectively, β = .33, β = .42). The second order factor accounted for 71% of the variance in Passion, 61% in Sacrifice, 76% in Identity, 62% in Pervasiveness, 44% in Purposefulness, 11% in Prosocial Orientation, and 18% in Transcendent Summons. Taken together, these results suggest that one second-order factor may not be the best higher order solution. Hence, Model 3 was estimated in which Transcendent Summons and Prosocial Orientation were allowed to correlate with the second order factor but were not allowed to load on it (Model 3: χ^2^(200) = 1213.11, CFI = .958, RMSEA = .055, 95% CI [.052 - .058], SRMR = .044). Results of this comparison are mixed. The chi-square test of close fit is below the chosen alpha level (Δχ^2^_(1)_ = 61.13, *p* < .001), and suggests to accept Model 3. Yet, differences in fit indexes show that changes in model fit are trivial and suggest to accept Model 2 for parsimony (ΔCFI = -.003; ΔRMSEA = .001; ΔSRMR = .006). As a further reason for accepting Model 2, we note that the chi square test may be unreliable when comparing models with a different number of factors, since the constraints imposed to the more complex model are at the limits of the parameter space [28]. Consistently with all other choices in this paper, we accepted Model 2, which is represented in Fig 1. These results support a multidimensional conceptualization of career calling structured in Passion, Sacrifice, Identity, Pervasiveness, Purposefulness, Transcendent Summons, and Prosocial Orientation. These last two factors are weakly related to calling and prevent the second order factor structure from being interpreted as a good multidimensional measurement framework of calling. We wondered whether these results represent actual properties of the true construct of calling rather than measurement artifacts, so we hypothesized that Transcendence Summons and Prosocial Orientation become part of calling during a later stage of its development. We tested this hypothesis estimating Model 2 on a sample of working adults (χ^2^(179) = 378.07, CFI = .918, RMSEA = .074, 95% CI [.063 - .084], SRMR = .06). Results showed that the loadings of Transcendence (.58, 95% CI [.45, .72]) and Prosocial Orientation (.69, 95% CI [.57, .81]) on the calling factor were significantly higher and almost twice the size of those estimated on students (respectively, .43, 95% CI [.38, .48] and .36, 95% CI [.30, .42]). In addition, loadings in the sample of working adults were above or very close to the commonly accepted threshold of .6. Results support the hypothesis that Prosocial Orientation and Transcendent Summons become part of calling during a later stage of its development.

### Invariance tests

#### Longitudinal invariance

Evidence of longitudinal measurement invariance is necessary before scores at either the single facets or the higher order construct can be compared across observations. We tested the longitudinal invariance of the second-order structure (Model 2) on students who took part to the three waves of data collection (*N* = 434). Identification of the mean structure was achieved constraining to zero the second-order factor mean and the first-order factor intercepts at Time 1. Seven of the items presented at Time 1 were modified before the second data collection, thus they were set as missing for the present analysis. Three of the deleted items saturated on the Identity factor; hence, Model 2 at Time 1 has six first-order factors. Table 3 presents fit indices and model comparisons. The second-order model showed a good fit to the data within each time point.

**Table 3:** Longitudinal measurement invariance test for Model 2.

	*χ*^*2*^	*df*	CFI	RMSEA	95% CI	SRMR	Δ*χ*^*2*^	Δ*df*	*p*	ΔCFI	ΔRMSEA	ΔSRMR
**Time 1**	205.5	84	.964	.058	.048 - .068							
**Time 2**	475.41	201	.957	.056	.05 - .063							
**Time 3**	436.31	201	.961	.055	.048 - .061							
**Configural**	3584.71	1575	.889	.054	.052-.057	.083						
**1st-order loadings**	3619.08	1600	.897	.054	.052-.056	.083	34.37	25	.10	.001	< .001	< .001
**2nd-order loadings**	3625.59	1611	.897	.054	.051-.056	.084	6.51	11	.84	< .001	< .001	-.001
**Item intercepts and factor means**	3675.59	1635	.896	.054	.051-.056	.084	50.00	24	.001	.001	< .001	< .001
**Item error variances**	3809.74	1672	.891	.054	.052-.057	.085	134.15	37	< .001	.005	< .001	-.001
**Factor variance**	3831.41	1685	.891	.054	.052-.056	.086	21.67	13	.061	< .001	< .001	-.001

*Note*. *N* = 434. Thresholds for accepting non-invariance: alpha level of the test of Δ*χ*^*2*^ < .005 and ΔCFI ≥ -.010, supplemented by ΔRMSEA ≥ .015 in RMSEA or ΔSRMR ≥ .010 (≥ .030 for testing metric invariance).

The fit of the configural invariance model was acceptable, meaning that the items loaded on the same first- and second-order factors across measurement occasions. According to the thresholds described in the previous section of the article, models testing the invariance of loadings, item intercepts, error variances and factor variances fit the data equally well than previous, less parsimonious, models. Hence, we can assume that they are all invariant across time.

#### Measurement invariance across different calling domains

Testing the absence of measurement bias in group comparisons is especially critical for the study of calling. Indeed, the assumption that a calling scale measures the same construct across different study domains may be too strong. Calling always has a target domain, and it is reasonable to wonder whether the experience of having a calling is qualitatively different for people interested in different domains. For example, Prosocial Orientation might be a key component of calling in a professional domain in which helping others is an important value, but this may not be true for domains in which self-interests are the modal motivation. To test multi-group measurement invariance of Model 2, domains with more than 100 participants in the students sample were selected (Psychology *n =* 248, Engineering *n =* 174, Medical Sciences *n =* 110). Results of measurement invariance analysis are reported in Table 4. Fitting the second-order model for each group separately yielded good fit indexes, so we can assume that configural invariance holds. Identification of the mean structure was achieved constraining to zero the second-order factor mean and the first-order factor intercepts of the Psychology group. Differences in chi-square, CFI, RMSEA and SRMR provide evidence of invariant first- and second-order factor loadings.

**Table 4:** Measurement invariance test across study domains for Model 2.

	χ^2^	*df*	CFI	RMSEA	95% CI	SRMR	Δχ^2^	Δ*df*	*p*	ΔCFI	ΔRMSEA	ΔSRMR
**Psychology**	416.91	201	.934	.066	.057-.075	.063						
**Engineering**	339.74	201	.944	.063	.051-.074	.059						
**Medical Science**	291.72	201	.945	.064	.047-.08	.07						
**Configural**	1048.36	603	.94	.065	.058-.071	.065						
**1st-order loadings**	1093.71	635	.938	.064	.057-.07	.071	45.34	32	.06	.002	.001	-.006
**2nd-order loadings**	1102.34	647	.939	.063	.057-.069	.069	8.63	12	.73	-.001	.001	-.003
**Item intercepts and factor means**	1214.55	677	.928	.067	.061-.073	.077	112.21	30	< .001	.011	-.004	-.003
**Item error variances**	1374.10	723	.912	.071	.066-.077	.083	159.56	46	< .001	.016	-.004	-.005
**Factor variance**	1417.97	737	.908	.072	.067-.078	.099	43.86	14	< .001	.004	-.001	-.016

*Note*. Thresholds for accepting non-invariance: alpha level of the test of Δ*χ*^*2*^ < .005 and ΔCFI ≥ -.010, supplemented by ΔRMSEA ≥ .015 in RMSEA or ΔSRMR ≥ .010 (≥ .030 for testing metric invariance).

The equality constraints on indicator intercepts and variances produced a significant loss of fit in terms of chi square and CFI, but a negligible decrease in RMSEA and SRMR. The equality constraints on factor variance produced a significant loss of fit in terms of chi square and SRMR, but a negligible decrease in CFI and RMSEA. In line with the thresholds adopted for model comparisons, our results suggest that loadings, item intercepts, error variances and factor variances are all invariant across study domains. Hence, the scale exhibited metric, scalar, and strict invariance.

### External validity

Regression analysis was performed to examine the relationship of each facet of calling with concurrent measures (having a vocation and calling orientation), and outcomes (living a calling, intention to continue studying and academic satisfaction) in our sample of students at Time 2. Results are reported in Table 5.

**Table 5:** External validity indices.

Calling facets	Vocation		Calling Orientation		Living a Calling		Intention to continue studying		Satisfaction	
	β	*f*^*2*^	β	*f*^*2*^	β	*f*^*2*^	β	*f*^*2*^	β	*f*^*2*^
**Prosocial Orientation**	.08**	.01	.16**	.03	.02	< .01	.01	< .01	-.05*	< .01
**Purposefulness**	.07*	.01	.10**	.01	.12**	.01	.09*	.01	.10**	.01
**Transcendent Summons**	.36**	.18	.01	< .01	.13**	.02	-.05	< .01	-.04	< .01
**Passion**	.10**	.01	.13**	.01	.33**	.08	.17**	.01	.62**	.27
**Sacrifice**	.04	.01	.08*	< .01	.07*	.01	.08*	< .01	.02	< .01
**Pervasiveness**	-.02	< .01	.07*	< .01	.002	< .01	-.05	< .01	-.06	< .01
**Identity**	.15**	.02	.08*	< .01	.10*	.01	-.003	< .01	-.07*	< .01
***N***	1603		1610		1258		1610		1607	
***R***^***2***^	.33		.19		.34		.05		.34	

Notes.

** *p* < .001

* *p* < .05. β represents standardized regression coefficients. *f*^*2*^ is a measure of effect size [30] that represent the proportion of variance uniquely accounted for by the predictor, over and above that of all other predictors in the model. Values denote small (*f*^*2*^≥.02), moderate (*f*^*2*^*≤*.15) and large (*f*^*2*^≥ .35) effects. Regression were computed within T2. Facets of calling represent composite scores.

The strongest predictor of having a vocation was Transcendent Summons, which accounted for 18% of the outcome variance not accounted for by other predictors in the model. Identity accounted for only 2% of the remaining variance. Prosocial Orientation, Passion and Purposefulness weakly predicted calling orientation [9]. The strongest predictor of living a calling was Passion for the study domain (*f*^*2*^ = .08), followed by Transcendent Summons that accounted for 2% of the residual variance. Passion also emerged as the strongest predictor of academic satisfaction, accounting for 27% of the variance not accounted for by other predictors. Sacrifice and passion were the stronger predictors of participants’ intention to continue their studies, although the effect sizes were small (*f*^*2*^ = .01).

To a varying extent, all facets of calling contributed to explain different correlates and outcomes of calling. Their effects varied in size from small to moderate, suggesting that facets of calling are not interchangeable: One facet of calling cannot be used in substitution of another facet nor of the higher-order calling construct, since it is likely that results obtained with one single facet will not generalize to other facets.

## Discussion

The accumulation of scientific knowledge on calling within a coherent corpus has been limited by the absence of a unified theoretical and measurement framework. The lack of a common theory and measurement scale may have limited the generalizability of results, and may have increased inconsistency of results across studies. This article proposes a unified model that combines the most common dimensions of calling and represents both the neoclassical and modern approaches to calling into a single theoretical and measurement framework. This model has been extensively tested against many kinds of bias. Consistently with most theoretical approaches [3, 31], results provide clear support for the multidimensionality of calling and suggest that both the neoclassical and modern approaches to calling tackle the same construct.

### The structure of calling

This paper presents initial empirical evidence that different models of calling may tackle the same construct. Yet, we found that the Transcendent Summons and Prosocial Orientation dimensions adequately represent the higher factor of calling in a sample of working adults and not in a sample of students. Relationships between these two facets and the other dimensions of calling are too low in the students sample to support a well-balanced multidimensional model. This result may suggest that the seven-facet structure of calling is developed later in life, and that working experiences are necessary to create consistency across calling dimensions. These results would be compatible with the hypothesis that only fully formed and mature callings include the Transcendent Summons and Prosocial Orientation dimensions. Alternatively, it may be that older people give more importance to typical neoclassical dimensions of calling than younger people. More research is needed to test these alternative interpretations. Also, our results may be limited to our specific samples. Indeed, in a sample of psychology students, Dik and colleagues [4] found correlations between Transcendent Summons, Prosocial Orientation and Purposefulness that are more in line with our sample of working adults than with our sample of college students. Finally, it is worth noting that Transcendent Summons and Prosocial Orientation are theoretically important to distinguish calling from different constructs, such as work passion [32]. If we define calling using only Passion, Pervasiveness, Purposefulness, Identity, and Sacrifice, the overlap is very large. Indeed, Vallerand and Houlfort [32] defined the construct of work passion as “a strong inclination toward an activity that people like, that they find important and in which they invest time and energy” (p. 175). This definition refers to aspects that are critical in the definition of calling: pleasure, meaningfulness, and willingness to sacrifice. Also, the authors suggest that one of the processes through which an activity becomes passion is the internalization of the activity within the individual’s identity. Finally, pervasiveness is involved in the distinction between harmonious and obsessive passion. The latter brings the individual to feel compelled to engage in the passionate activity, and this causes conflicts with other areas in the person’s life [33]. Thus, the two facets that allow distinguishing calling from work passion are Transcendent Summons and Prosocial Orientation.

### Measurement invariance of the seven facets and the higher order construct

Any comparison of the same construct across time or groups assumes that the measures are invariant. Measurement invariance is probably the most important question to address before any comparison between groups or across time points is conducted. Indeed, the assumption of measurement invariance may not always be met. When noninvariance is present, score comparisons represent measurement bias rather than theoretically interpretable empirical evidence, unless sophisticated statistical models are used to remove bias. We tested the assumption of invariance across calling domains and time points. Our findings suggest that the scale is longitudinally invariant at the strict level. Hence, scores at the UMCS can be meaningfully compared across time as they reflect changes in true scores rather than measurement artifacts. Measurement invariance across domains may be even more critical for the study of calling, which always has a specific target domain. In this study, we investigated the measurement invariance of the UMCS across the study domains of medical science, engineering, and psychology. The UMCS was found to be invariant at the configural and metric levels across groups. Hence, our results suggest that the structure of calling is independent from the domain of the call. Relations between first and second order factors, and relations between our measure of calling and other external variables can be compared across groups, even using composite scores. The multi-group invariance analysis yielded evidence of strict invariance. Scores at the UMCS can be meaningfully compared across calling domains because group differences in estimated factor means will be unbiased [33; 34].

### External validity

Overall, the UMCS showed good external validity. The seven facets of calling accounts for 33% and 19% of the variance of two one-item measures of calling, 34% of the variance in living a calling and academic satisfaction, and only 5% of the dropout measure. Remarkably, facets of calling differently contribute to these relationships, with effects that vary from 0% to 27%. Passion best predicts living a calling and academic satisfaction. Transcendent Summons and Identity are mostly related to a one-item measure assessing participants’ belief of having a vocation. Prosocial Orientation and Purposefulness are both, yet weakly, related to Wrzesniewski and colleagues’ [9] calling measure. These results suggest that there is no single facet that predicts all outcomes of calling. Although researchers interested in predicting a specific outcome may effectively employ a smaller subset of facets, generalizing these results to the higher-order construct of calling would probably be unwarranted.

## Conclusions

In this paper, we provided the first, although initial, evidence that two alternative theoretical accounts of calling actually tackle the same construct. Yet, more research is needed on the unified 7-facet model of calling that we employed in our studies. Indeed, we found that the second-order factor structure holds well in a sample of working adults, but not in a sample of college students. There are many possible accounts for this result, such as that the factor structure of calling changes after first work socialization. We hope future research will help to shed light on this topic. In terms of validity, our results are critically important for the study of calling because they suggest that mean composite scores at the UMCS, including correlations among them, can be compared longitudinally and across calling domains, providing support for previous and future studies without a direct test of this fundamental assumption. Finally, we found that different facets of calling have different correlates and outcomes. This result suggests using caution when generalizing the results of research that employs a subset of the seven-facet model to the higher-order construct of calling. What may be true for a model of calling may not be true for a different model. Using the 22-item scale that we extensively tested in this paper may help researchers to enhance the generalizability of their results.
